# Geographic analysis of low birthweight and infant mortality in Michigan using automated zoning methodology

**DOI:** 10.1186/1476-072X-8-10

**Published:** 2009-02-18

**Authors:** Sue C Grady, Helen Enander

**Affiliations:** 1 Department of Geography, 130 Geography Building, Michigan State University, East Lansing, Michigan 48824, USA; 2 Department of Geography, 1H Geography Building, Michigan State University, East Lansing, Michigan 48824, USA

## Abstract

**Background:**

Infant mortality is a major public health problem in the State of Michigan and the United States. The primary adverse reproductive outcome underlying infant mortality is low birthweight. Visualizing and exploring the spatial patterns of low birthweight and infant mortality rates and standardized incidence and mortality ratios is important for generating mechanistic hypotheses, targeting high-risk neighborhoods for monitoring and implementing maternal and child health intervention and prevention programs and evaluating the need for health care services. This study investigates the spatial patterns of low birthweight and infant mortality in the State of Michigan using automated zone matching (AZM) methodology and minimum case and population threshold recommendations provided by the National Center for Health Statistics and the US Census Bureau to calculate stable rates and standardized incidence and mortality ratios at the Zip Code (n = 896) level. The results from this analysis are validated using SaTScan. Vital statistics birth (n = 370,587) and linked infant death (n = 2,972) records obtained from the Michigan Department of Community Health and aggregated for the years 2004 to 2006 are utilized.

**Results:**

For a majority of Zip Codes the relative standard errors (RSEs) of rates calculated prior to AZM were greater than 20%. Spurious results were the result of too few case and birth counts. Applying AZM with a target population of 25 cases and minimum threshold of 20 cases resulted in the reconstruction of zones with at least 50 births and RSEs of rates 20–22% and below respectively, demonstrating the stability reliability of these new estimates. Other AZM parameters included homogeneity constraints on maternal race and maximum shape compactness of zones to minimize potential confounding. AZM identified areas with elevated low birthweight and infant mortality rates and standardized incidence and mortality ratios. Most but not all of these areas were also detected by SaTScan.

**Conclusion:**

Understanding the spatial patterns of low birthweight and infant deaths in Michigan was an important first step in conducting a geographic evaluation of the State's reported high infant mortality rates. AZM proved to be a useful tool for visualizing and exploring the spatial patterns of low birthweight and infant deaths for public health surveillance. Future research should also consider AZM as a tool for health services research.

## Introduction

Infant mortality refers to infants born alive who die within their first year of life. In 2006, Michigan's infant mortality rate was 7.6 infant deaths per 1,000 live births with African American infants at substantially higher risk (17.7) than white infants (5.2) of death [1]. The primary adverse reproductive outcomes that increase newborn's risk of death are premature birth (i.e., infants born less than 37 weeks gestation), low birthweight (i.e., infants born less than 2,500 grams), which includes very low birthweight (i.e., infants born less than 1,500 grams) and congenital defects [1]. Infants born prematurely, low birthweight and/or with congenital defects are at increased risk of death because of undeveloped or poorly developed organs and/or organ systems and the inability to physiologically respond to their external environment. High-risk neonates (i.e., infants less than 1 month of age) also require high quality perinatal-neonatal health care and without supportive medical intervention are at increased risk of mortality.

The purposes of this study are to (a) visualize and explore the spatial patterns of low birthweight and infant mortality in the State of Michigan using automated zone matching (AZM) tool [2] for automated zone design, and (b) to evaluate AZM as a potential tool for public health surveillance. AZM contains a computationally intensive algorithm that recombines geographic units from a large spatial dataset into a smaller set of output zones from within which to calculate stable rates and ratios. This recombination is an iterative process, by which one geographic unit is randomly selected and user defined attribute constraint parameter(s) are evaluated (e.g., target population (TP)). If this parameter is not met AZM will search contiguous units until it is achieved, thereafter, aggregating the data and dissolving internal boundaries to create a new zone. This "initial random aggregation" (IRA) will serve as the basis for subsequent iterative decisions. The TP parameter is evaluated by summing the sum of squared differences between the target and actual population counts. The zone design with the smallest TP value is generally considered optimal. Two additional parameters that aim to maximize homogeneity within zones and heterogeneity between zones include shape and homogeneity constraints. The shape constraint minimizes the shape of zones (i.e., maximizes shape compactness) and is calculated using a simple shape statistic (perimeter^2^/area) (P2A). The zone design with the lowest overall P2A value, derived from summarizing each of the local P2A values is considered optimal. Homogeneity constraints maximize the similarity of user-defined characteristics of the target population and/or features of the local environment in the aggregation process. The degree of similarity is evaluated using the intra-area correlation (IAC) coefficient. The zone design with the strongest overall IAC coefficient, derived from summarizing all of the local IAC coefficients is considered optimal. A more in-depth discussion about the functionality of AZM and parameter estimation for optimal zone design is provided in the background and methods sections of this paper.

In this study we utilize AZM as a surveillance tool to create stable rate maps of low birthweight incidence (hereafter, referred to as low birthweight rates) and infant mortality and standardized incidence ratios (SIRs) and standardized mortality ratios (SMRs) constructed at the Zip Code level. Understanding the spatial patterns of low birthweight and infant mortality are important for generating mechanistic hypotheses and targeting high-risk areas for public health intervention and prevention programs and health care service needs. The National Vital Statistics Reports (NVSR) [3] and Center for Disease Control and Prevention (CDC) [4] define stable rates as those with at least 20 cases in the numerator, which corresponds to a relative standard error (RSE) of 22%, respectively, and a population denominator of at least 50 (unweighted) [5]. The RSE is the standard error as a percent of the rate itself. For example, a RSE of 25% means that the standard error is one-quarter the size of the rate. In this analysis we use 25 as the target number of cases and 20 as the minimum case threshold. We use a target number of cases instead of a target population in the estimation of stable rates because population-based thresholds alone can mask low case counts resulting in artificially low rates with large relative standard errors. In addition, a simple shape constraint (P2A) is applied to encourage the creation of compact zones. Compact zones are important because they will minimize potential confounding in future mechanistic studies. Relatedly, we use maternal race (i.e., African American versus all other racial and ethnic groups) as our homogeneity constraint in order maximize maternal and infant homogeneity by race within zones and heterogeneity between zones. These constraints will be used to capture spatial-racial disparities in low birthweight and infant mortality in Michigan.

Following the recombination of Zip Codes into an optimal zone design the low birthweight and infant mortality rates are calculated using the sum of the number of low birthweight cases or infant deaths in the numerator and all live births in the denominator. The number of births within each new zone is also used to calculate SIRs and SMRs using the indirect method of standardization. Tables of the rates and ratios calculated by zone are then joined to the new zone geography and thematic maps are created to visualize and explore their spatial patterns. In this study, we explore the sensitivity of the rates and ratios to different zoning systems by comparing the P2A, IAC and TP results from 50 random restarts on the same data. We also validate the spatial patterns derived from optimal zone design by comparing the rates and ratios with spatial clusters detected in SaTScan using a Poisson-based modeling approach.

In Michigan there are 15 metropolitan areas located in the approximate lower third of the State. The upper two-third of Michigan is relatively rural with the population density in the Upper Peninsula about 19 persons per square mile [6]. Low birthweight and infant mortality rates calculated at the Zip Code level for upper Michigan, particularly in the Upper Peninsula, are therefore likely to be unstable. In addition, Michigan has a high level of racial residential segregation with African Americans living primarily in metropolitan principal cities ^(1) ^and the city of Detroit where low birthweight and infant mortality rates also calculated at the Zip Code level are likely to be stable. These spatial differentials in rate stability and instability in low birthweight and infant mortality by race could lead to spurious interpretations of race-specific risk and place-based risk factors, which could result in misguided hypothetical mechanisms underlying racial disparities in these outcomes. This research using AZM to stabilize the low birthweight and infant mortality rates and ratios in Michigan is therefore warranted.

^(1) ^Principal cities are defined as cities, villages or Census Designated Places that meet criteria involving number of people and relative number of in-commuters and out-commuters. This term replaces the term "central city." In Michigan, 2003 there were 31 principal cities in metropolitan areas (population > 50,000) and 16 principal cities in micropolitan areas (population > 10,000 and < 50,000) (Library of Michigan, [6]).

## Background

There are a small but growing number of studies utilizing zone design methodologies to evaluate the spatial patterns of health outcomes and local risk factors. Cockings and Martin [7] conducted seminal research using AZM to measure the correlation between self-reported long-term illness (LLTI) and area-level deprivation at the enumeration district (ED) (n = 1,970) and wards (n = 177) scales in Avon, a former county in the United Kingdom. This analysis was conducted at two scales to assess potential bias associated with the modifiable areal unit problem (MAUP) [8] scale effect. LLTI was measured using standardized morbidity ratios derived from an indirect method of standardization (i.e., estimating the expected number of cases by multiplying age-specific population counts by country-wide age-specific LLTI rates and dividing the expected by the observed number of cases). Area-level deprivation was measured using the Townsend score (i.e., the proportion of people without a car, households in overcrowded accommodations, households not owner-occupied and unemployment). Pearson correlation coefficients were estimated. The AZM parameters included a target population constraint of 250 people, aged 0–64 years, or a minimum population threshold constraint of 90% the target value. AZM runs were repeated with increasing target population counts in 250 increments up to 4,500 people to cover the population range in EDs and wards (total 13 runs). The shape of output zones were defined by a simple shape statistic (P2A). No homogeneity constraints were used in this analysis. The authors implemented 50 random restarts for each set of population and shape constraints and within each zone standardized morbidity ratios and area level deprivation scores were calculated and correlation coefficients were estimated. The results from these analyses were also compared with LLTI-deprivation correlation coefficients estimated using the ED and ward boundaries. This study found that with increasing scale (i.e., increasing population thresholds) the LLTI-deprivation correlation coefficients also increased, which was a finding similar to previous research [8]. Optimal zone design had an LLTI-deprivation correlation coefficient of 0.88 at a target population of 3,750 people (mean population size 4,291 people), which was most closely related to the ward scale of analysis (mean population size 4,364). The LLTI-deprivation correlation coefficient at the ED scale was 0.72 and the ward scale 0.86. While the coefficients results for AZM zones and wards were fairly similar (0.88 versus 0.86) the population within wards were extremely variable (e.g., ward range, 14,290 people compared to AZM zone range, 3,000 people), with large differences between rural and urban areas. The authors conclude that the population stability derived from AZM resulted in more reliable LLTI-deprivation correlation coefficients than those based on ward boundaries, demonstrating the importance of AZM in this analysis.

A study by Haynes et al [9] used A2Z software [10,11] to explore the similarity between neighborhoods derived from zone design and those relayed subjectively by local government officers and planners in the city of Bristol, United Kingdom. Seven zone designs were constructed from 814 EDs using the homogeneity constraint "material deprivation" defined by the Townsend score and other different parameters. Specifically, zone designs 1–3 used increasingly strong shape constraints. The weakest shape constraint "prevented long linear strings of areas from being joined by checking the graphic network structure for each zone." The medium shape constraint minimized local spatial dispersion after five "uncontrolled" iterations [9]. The strongest shape constraint used the P2A parameter in the initial random aggregation. The fourth zone design used a shape constraint that enforced the separation of EDs across railways and major roads by giving pairs of EDs on either side of major routes an adjacency value of zero versus one for non-contiguous EDs. The fifth zone design aligned zone boundaries along wards using similar mean Townsend scores. The sixth zone design used a weak shape constraint as in zone design one, but maximized the homogeneity of housing type (i.e., proportion of households detached, semi-detached and terraced houses, purpose-built flats and converted flats). The seventh zone design was similar to the sixth but also tried to align zone boundaries with ward boundaries. The evaluation of optimal zone design was that with the strongest IAC and zone shapes and sizes similar to that of EDs. The EDs were considered the standard from which to measure the similarity between zones and EDs using a "similarity index" with 1 representing identical boundaries and zero representing no common boundaries. This study found that AZM zone design one was much less compact in shape than EDs and had only moderate "similarity." Zone designs 2–5 were progressively more similar to EDs and more compact in shape, but increasingly less homogeneous. Zone designs 6 and 7 preformed the "best" because of their strong IAC in terms of semi-detached housing and common boundaries. The authors concluded that in the construction of optimal zone designs there needs to be balance between the use of neighborhood homogeneity constraints and constraints relating to zone shape and boundary alignment.

Flowerdew et al [12] used automated zone design (AZTool system formerly AZM) [13] to explore the MAUP [8] scale and zone effects in the district of Swindon, England using population data and data on LLTI and demographic-social factors in EDs (n = 1,268) and wards (n = 148). The parameters were a target population of 8,136 people, corresponding to the average population of existing wards, and a minimum population threshold of 2,651, corresponding to the population size of the smallest ward. The zone design was based on six criteria: contiguous zones, no zone should be entirely surrounded by another, the number, shape and size of zones should be similar to the number, shape and size of wards and zones should have strong internal homogeneity. The optimal zone design was considered in order of priority the TP, P2A, and IAC values. The scale effect was evaluated by measuring the difference in population homogeneity (i.e., the IAC of LLTI-demographic-social factors) in zones compared to EDs and wards. This analysis showed that the IAC differed for different demographic-social factors at these different scales. For example, at the ED scale the percentage of people of pensionable age was most strongly correlated with LLTI, whereas, at the ward scale the percentage of male unemployment was most strongly correlated with LLTI, controlling for other risk factors. The MAUP zone effect was also evaluated using the IAC in addition to multiple regression to estimate the effect of known and suspected risk factors on the percent of population with LLTI. This analysis showed that the correlation between LLTI and various demographic-social variables varied for different zone designs; however, the direction of the estimate remained the same for most but not all variables, demonstrating a zone effect. In summary, the authors found that zone designs that emphasized the TP and P2A constraints were most optimal and there seemed to be little benefit from the use of homogeneity constraints.

Stafford et al [14] used zone design methods (ZDES 3b software) [15] to create neighborhoods in the London boroughs of Camden and Islington based on socioeconomic homogeneity constraints, defined as the proportion of residents living in rented social housing and physical boundaries based on roads and railways to explore the effect of these neighborhood characteristics on the health outcomes body mass index, alcohol intake, exercise, smoking behavior and self-rated general health, controlling for individual level characteristics. The two zone designs were similar in number to the real census wards (n = 34). Two-level hierarchical models were implemented to estimate the variation in health outcomes within and between zones in each zone design. Similar models were implemented in wards for comparison purposes. Individual-level characteristics were modeled at level-1 and the intercept and slope coefficients were modeled at level-2. The results showed positive and significant associations for alcohol intake, walking, smoking and self-rated health using all three boundary definitions. The strongest association was between alcohol intake and housing tenure using the boundaries defined with the homogeneity constraint housing tenure. However, the magnitude of the between-zone variation was small in comparison to the within-variation (i.e., individual-level variation) for all boundary definitions suggesting that the two zone designs had no substantial advantage over the ward boundaries. In contrast, a study conducted by Riva et al [16] showed that census tract administrative boundaries were limited in their ability to measure active living potential (i.e., environments that were conducive for walking). Through the use of zone design, using the homogeneity constraints population density, land use mix and accessibility to services, the authors identified seven types of environments within which, varying levels of active living were possible.

These aforementioned studies compliment two existing but separate bodies of health research that could be affected by MAUP scale and/or zoning effects. The first body of research applies geographic methodologies to correct for rate instability and/or preserve case confidentiality through data suppression [3]. The most commonly used methods to address these concerns are probability mapping [17], spatial filtering [18,19], Bayesian smoothing [20], and cluster detection methods such as SaTScan [21,22]. The second body of research evaluates neighborhood effects on health outcomes [23-32] in which neighborhood risk characteristics are explored. This study complements these two existing bodies of research by providing an empirical example how AZM can be used as a surveillance tool to define the spatial patterns of low birthweight and infant mortality in the State of Michigan. From these spatial patterns meaning may be derived about their underlying processes (e.g., underlying mechanisms), including individual and local environmental risk factors and access to health care services.

**Figure 1:**
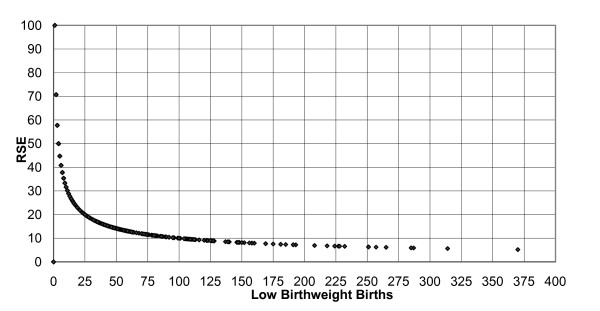
**Plot of relative standard errors by number of low birthweight births, Michigan 2004 to 2006**.

This study implements the NVSR [3] guidelines to use at least 20 cases in the numerator and 50 cases [5] in the denominator to estimate stable low birthweight and infant mortality rates and ratios. Figure 1 displays the relationship between low birthweight cases and the corresponding RSE to show how the numeric value of 20 cases was derived.

The methodologies to calculate RSEs and confidence intervals to validate the stability of the rates and ratios will be based on the underlying distributions of births and deaths. The NVSR [33] reports that vital statistics birth and linked infant death records "include a complete case count because more than 99% of all births and deaths in the United States are registered." "These data are not subject to sampling error but they may be affected by nonsampling error in the registration process, particularly involving missing case information such as Zip Code of residence" [33]. The number of births and deaths that actually occur are thought of as "one outcome in a series of possible outcomes under the same (or similar) circumstances" and the numbers of births and deaths are therefore, subject to random variation. When the numbers of births or deaths is large the distribution is assumed to follow a normal distribution and when the number of births is small (i.e., less than 100) the distribution is assumed to follow a Poisson distribution. The RSE of normally distributed data will be small, while the RSE of Poisson distributed data is likely to be large. This study will utilize the methodologies provided by the NVSR [3,33] to calculate the RSEs and confidence intervals for rates and ratios based on these two distributions. RSEs and confidence intervals will be the mechanisms by which the stability of rates and ratios derived from AZM zone definitions are evaluated.

## Results

### Descriptive Statistics

From 2004 to 2006 there were 370,587 live singleton births in Michigan. Of these, 23,761 (6.4%) infants were born low birthweight and 2,972 infants died before their first birthday (Table 1). The rate of low birthweight for African American infants was 12.2 per 100 live births and white infants 5.1 per 100 live births, risk ratio (RR) = 2.3. Thus, African American infants were over twice as likely to be born low birthweight than white infants. The overall infant mortality rate was 8.0 deaths per 1,000 live births. The mortality rate for African American infants was 17.0 per 1000 live births compared to white infants 6.1, RR = 2.7. Thus, African American infants were almost three times as likely to die before their first birthday as white infants. The mortality rate among African American infants born low birthweight was 11.2 per 1,000 live births compared to 8.2 per 1,000 live births for white infants, RR = 1.36. The large disparities in low birthweight and infant mortality by race demonstrate the importance of using this maternal characteristic as a homogeneity constraint in the creation of stable rate and ratio maps. Mother's education was not used as a homogeneity constraint because of the large number of birth records with missing data. However, the data available show that as maternal education increased the rate of low birthweight and infant mortality declined. For example, the low birthweight rate among infants born to mothers with less than a high school education was 9.1 per 100 live births compared to 7.1 for mothers with a high school education and 5.0 for mothers with a college education. Likewise, the mortality rate among infants of mothers with less than a high school education was 10.7 compared to 9.6 for mothers with a high school education and 6.1 for college-educated mothers. These findings show a strong inverse relationship between maternal education and low birthweight and maternal education and infant mortality.

**Table 1:** Descriptive statistics of low birthweight and infant deaths, Michigan 2004–2006.

	All Births		Low Birthweight		Deaths		Low Birthweight Incidence	Infant Mortality
	(n = 370587)		(n = 23761)		(n = 2972)			

	No.	(%)	No.	(%)	No.	(%)	Rate^(3)^	Rate^(4)^

Infant Sex								
Male	189898	51.2	11307	47.6	1637	55.42	6.0	8.6
Female	180676	48.7	12445	52.4	1317	44.58	6.9	7.3
Missing^(1)^	13		9		18			

Mother's Race								

White	287624	78.2	14695	62.3	1757	59.7	5.1	6.1
Black	64986	17.7	7928	33.6	1105	37.6	12.2	17.0
American Indian	2073	0.6	109	0.5	23	0.8	5.3	11.1
Other^(2)^	13259	3.6	858	3.6	58	1.9	1.9	1.1
Missing	2645		171		29			

Mother's Education								

Less than High School	63227	18.5	5722	26.1	679	24.5	9.1	10.7
High School	113708	33.4	8092	36.9	1090	39.4	7.1	9.6
College	163850	48.1	8131	37.1	999	36.1	5.0	6.1
Missing	29802		1816		204			

^(1) ^Data variable missing from dataset

^(2) ^American Indian, Asian and Hawaiian and Pacific Islanders

^(3) ^Number of low birthweight births per 100 live births

^(4) ^Number of infant deaths per 1,000 live births

**Figure 2:**
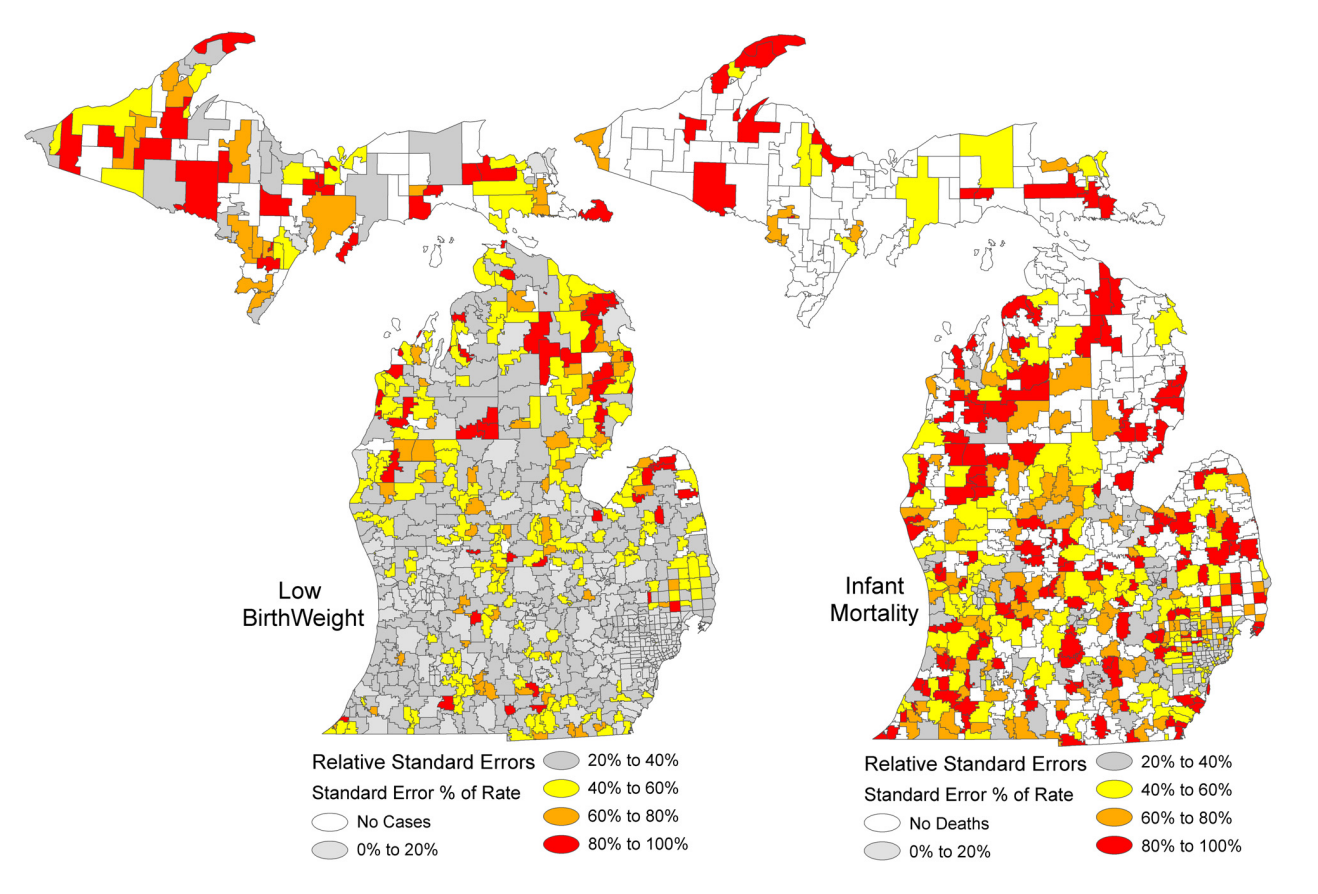
**Maps of relative standard errors of low birthweight incidence rates and infant mortality rates before AZM, Michigan 2004 to 2006**.

There were 896 Zip Codes used in this analysis. Of these, 65.2% of Zip Codes had less than 20 low birthweight births and 60.7% of Zip Codes had less than 20 infant deaths. These findings demonstrate that using Zip Codes as the unit of analysis will result in a high proportion of unreliable rate and ratio estimates. Figure 2 shows the spatial patterns of the RSEs for low birtweight rates in Michigan using the Zip Code boundaries. The findings were as expected with large RSEs of rates in Zip Codes in Upper Michigan where population density is low compared to Lower Michigan. In Lower Michigan there are a few scattered Zip Codes with large RSEs located outside of metropolitan principal cities. The map of infant mortality rates shows a substantial number of Zip Codes with large RSEs throughout the state and lower but still elevated RSEs in the metropolitan areas; demonstrating that for rare events such as infant deaths, the use of Zip Codes as a unit of analysis to calculate rates and ratios will result in a substantial number of unstable estimates across the state.

### Automated Zoning Methodology

Following AZM the number of Zip Codes was reduced from 896 to 375 zones for low birthweight and 98 zones for infant deaths. The low birthweight rates and SIRs and infant mortality rates and SMRs were calculated within the optimal zone designs for these outcomes, each zone having at least 20 cases/deaths and 50 births; therefore, meeting case [3] and population [5] thresholds for stable rates and ratios. In this study the optimal zone design was determined by ranking the P2A and IAC output results and then comparing those two zone designs that ranked highest (i.e., had the smallest P2A and strongest IAC). We also compared the IRA with these two "best" zone designs. Since the IRA zone design was very similar to the two "best" zone designs we decided to use the IRA to calculate the rates and ratios. When AZM is run on multiple occasions using the same data, initial settings and number of iterations the same IRA zone configuration will result. Thus for public health surveillance purposes the IRA would be the optimal zone design because it has stable geography from within which to monitor and evaluate rates and ratios over time.

**Figure 3:**
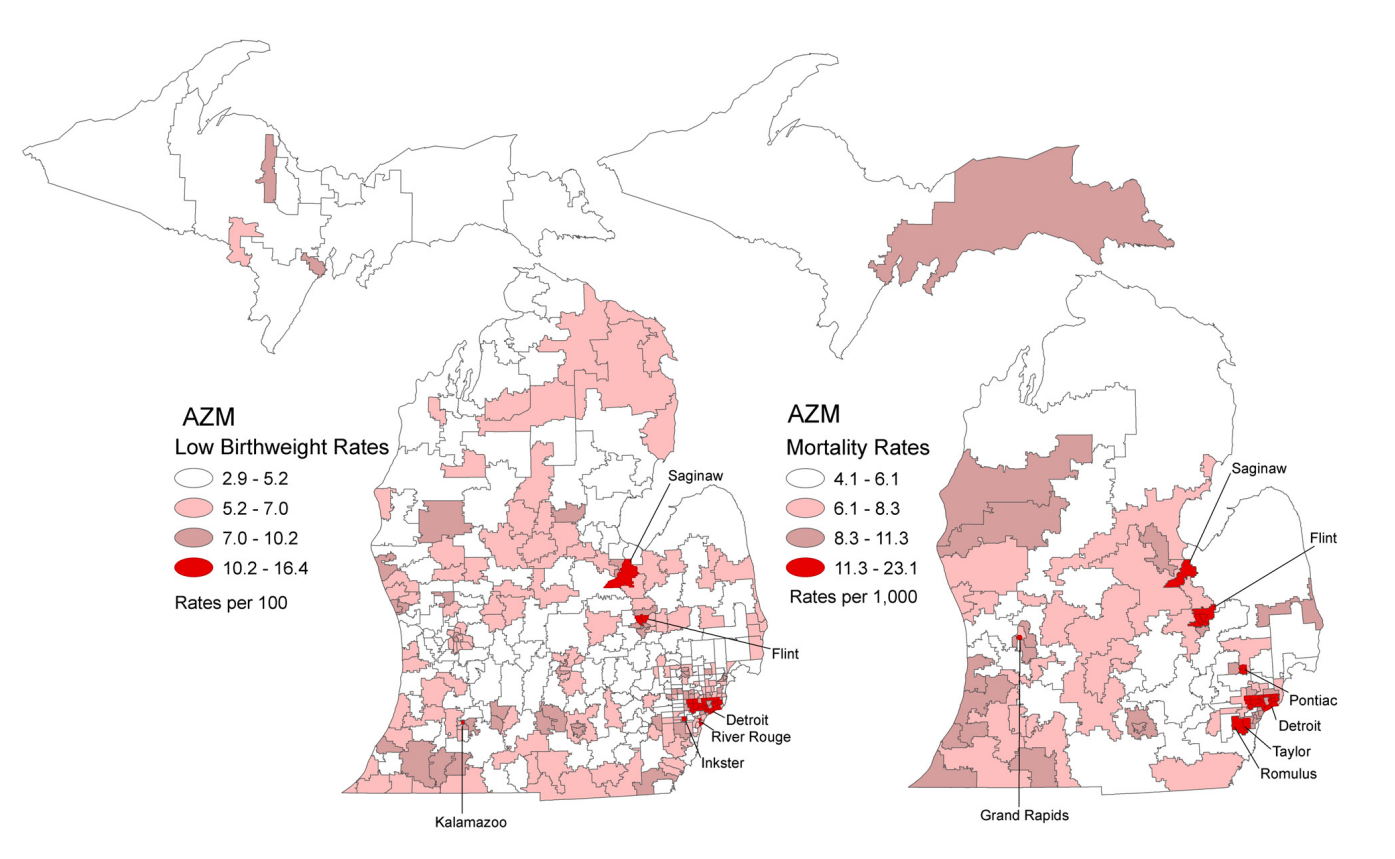
**Maps of low birthweight incidence rates and infant mortality rates following AZM, Michigan 2004 to 2006**.

Figure 3 shows maps of stable low birthweight and infant mortality rates using their optimal zone designs. The spatial patterns show high rates of low birthweight scattered throughout the state. The highest rate of low birthweight, 16.4 per 100 live births (95% CI 15.4, 17.5) is in the principal metropolitan city of Detroit (Table 2). Low birthweight rates were also very high (i.e., > 10.2) in Flint, Inkster, Kalamazoo, River Rouge and Saginaw. Those zones with infant mortality rates between 11.3 and 23.0 per 1,000 live births were located in Detroit and other metropolitan cities including Flint, Grand Rapids, Pontiac, Romulus, Saginaw and Taylor. The highest infant death rate, 23.1 deaths per 1,000 live births (95% CI 15.6, 33.0) was located in the western portion of Detroit (Table 3). These spatial patterns in low birthweight and infant mortality show discrepancies with low to moderate rates of low birthweight yet high infant deaths in Grand Rapids, Pontiac, Romulus and Taylor. These findings suggest that future investigations should target these areas to explore causes of death and health care services coverage. Relatedly, Inkster, Kalamazoo and River Rouge have elevated rates of low birthweight but relatively low rates of infant mortality suggestive of protective mechanisms in these areas.

**Table 2:** AZM Zones with the highest low birthweight rates and standardized incidence ratios, Michigan 2004-2006.

Zone	LBW Cases	Births	Rate^a^	95% CI	SIR^b^	95% CI	RSE
298	160	972	16.4	15.4, 17.5	2.5	2.1, 2.9	7.9
295	105	722	14.5	13.4, 15.6	2.2	1.8, 2.7	9.7
318	44	303	14.5	10.5, 19.4	2.2	1.5, 2.9	15.0
288	265	1839	14.4	13.7, 15.0	2.2	1.9, 2.5	6.1
96	185	1297	14.2	13.4, 15.0	2.2	1.9, 2.5	7.3
282	256	1825	14.0	13.3, 14.6	2.1	1.9, 2.4	6.2
359	225	1605	14.0	13.3, 14.7	2.1	1.9, 2.4	6.6
283	224	1625	13.7	13.1, 14.4	2.1	1.8, 2.4	6.6
284	291	2214	13.1	12.5, 13.6	2.0	1.8, 2.2	5.8
111	73	561	13.0	10.1, 16.3	2.0	1.5, 2.4	11.7
95	286	2236	12.7	12.2, 13.3	1.9	1.7, 2.2	5.9
290	223	1764	12.6	12.0, 13.2	1.9	1.7, 2.2	6.6
281	365	2902	12.5	12.1, 13.0	1.9	1.7, 2.1	5.2
297	149	1216	12.2	11.5, 12.9	1.9	1.6, 2.2	8.1
360	220	1796	12.2	11.6, 12.8	1.9	1.6, 2.1	6.7
91	255	2083	12.2	11.7, 12.7	1.9	1.6, 2.1	6.2
300	194	1588	12.2	11.6, 12.8	1.9	1.6, 2.1	7.1
304	75	615	12.1	9.5, 15.2	1.9	1.4, 2.3	11.5
302	92	758	12.1	9.7, 14.8	1.8	1.5, 2.2	10.4
303	61	504	12.1	9.2, 15.5	1.8	1.4, 2.3	12.8
90	203	1691	12.0	11.4, 12.5	1.8	1.6, 2.1	7.0
296	87	770	11.2	9.0, 13.9	1.7	1.3, 2.1	10.7
106	145	1286	11.2	10.6, 11.8	1.7	1.4, 2.0	8.3
161	255	2313	11.0	10.5, 11.4	1.7	1.5, 1.9	6.2
301	320	2928	10.9	10.5, 11.3	1.7	1.5, 1.8	5.5
324	52	484	10.7	8.0, 14.0	1.6	1.2, 2.1	13.8
89	139	1356	10.2	9.7, 10.7	1.5	1.3, 1,8	8.4
80	107	1051	10.1	9.5, 10.7	1.5	1.2, 1.8	9.6
361	127	1254	10.1	9.5, 10.6	1.5	1.3, 1.8	8.8
292	51	505	10.0	7.5, 13.2	1.5	1.1, 2.0	14.0
286	232	2326	9.9	9.5, 10.3	1.5	1.3, 1.7	6.5
243	119	1204	9.8	9.3, 10.4	1.5	1.2, 1.8	9.1
244	78	802	9.7	7.6, 12.1	1.5	1.1, 1.8	11.3
320	37	383	9.6	6.8, 13.3	1.5	1.0, 1.9	16.4
85	62	674	9.1	7.0, 11.7	1.4	1.0, 1.7	12.7
251	62	674	9.1	7.0, 11.7	1.4	1.0, 1.7	12.7
43	63	685	9.1	7.0, 11.7	1.4	1.0, 1.7	12.5
77	69	787	8.7	6.8, 11.0	1.3	1.0, 1.6	12.0
317	97	1137	8.5	6.9, 10.4	1.3	1.0, 1.5	10.1
294	115	1366	8.4	7.9, 8.8	1.3	1.0, 1.5	9.3
33	81	963	8.4	6.6, 10.4	1.3	1.0, 1.5	11.1
338	158	1800	8.3	7.9, 8.7	1.2	1.0, 1.5	8.1
193	158	2016	7.8	7.4, 8.1	1.2	1.0, 1.4	7.9
311	160	2043	7.8	7.4, 8.1	1.2	1.0, 1.4	7.9
258	140	1815	7.7	7.3, 8.0	1.2	1.0, 1.4	8.4
105	112	1454	7.7	7.3, 8.0	1.2	0.9, 1.4	9.4

Abbreviations: LBW, Low Birthweight; SIR Standardized Incidence Ratio; CI, Confidence Interval; RSE, Relative Standard Error

^a ^Rate is per 100 live births; ^b ^Standard Population of Michigan, 2000 incidence ratios, Michigan, 2004–2006.

**Table 3:** Zones with the highest infant mortality rates and standardized mortality ratios, Michigan, 2004–2006.

Zone	Deaths	Births	Rate^a^	95% CI	SMR^b^	95% CI	RSE
25	30	1297	23.1	15.6, 33.0	2.8	1.8, 3.9	18.4
75	37	1694	21.8	15.3, 30.1	2.7	1.8, 3.6	16.6
74	39	1839	21.2	15.0, 28.9	2.6	1.8, 3.4	16.8
77	45	2490	18.1	13.1, 24.1	2.2	1.6, 2.9	15.0
21	29	1625	17.8	11.9, 25.6	2.2	1.4, 3.0	18.7
78	28	1588	17.6	11.7, 25.4	2.2	1.3, 3.0	19.0
71	51	2902	17.6	13.0, 23.1	2.1	1.5, 2.7	14.1
79	50	2928	17.1	12.6, 22.5	2.1	1.5, 2.7	14.2
22	52	3047	17.1	12.7, 22.3	2.1	1.5, 2.7	13.9
82	28	1669	16.8	11.1, 24.2	2.0	1.3, 2.8	19.0
72	30	1825	16.4	11.0, 23.4	2.0	1.3, 2.7	18.4
8	31	1939	16.0	10.8, 22.6	1.9	1.2, 2.7	18.1
67	44	2773	15.9	15.0, 11.5	1.9	1.3, 2.5	15.1
95	25	1605	15.6	10.0, 22.9	1.9	1.1, 2.7	20.1
23	32	2083	15.4	10.5, 21.6	1.9	1.2, 2.5	17.8
96	25	1796	13.9	9.0, 20.5	1.7	1.0, 2.4	20.1
3	30	2191	13.7	9.2, 19.5	1.7	1.1, 2.3	18.3
24	54	4000	13.5	10.1, 17.6	1.6	1.2, 2.1	13.6
65	26	2006	13.0	8.4, 18.9	1.6	1.0, 2.2	19.7
32	25	1989	12.6	8.1, 18.5	1.5	0.9, 2.1	20.1
55	32	2638	12.1	8.2, 17.1	1.5	0.9, 2.0	17.7
87	30	2488	12.1	8.1, 17.2	1.5	0.9, 2.0	18.3
62	20	1704	11.7	7.1, 18.1	1.4	0.8, 2.1	22.4

Abbreviations: SMR, Standardized Mortality Ratio; CI, Confidence Interval; RSE, Relative Standard Error

^a ^Rate is per 1,000 live births; ^b ^Standard Population of Michigan, 2000

**Figure 4:**
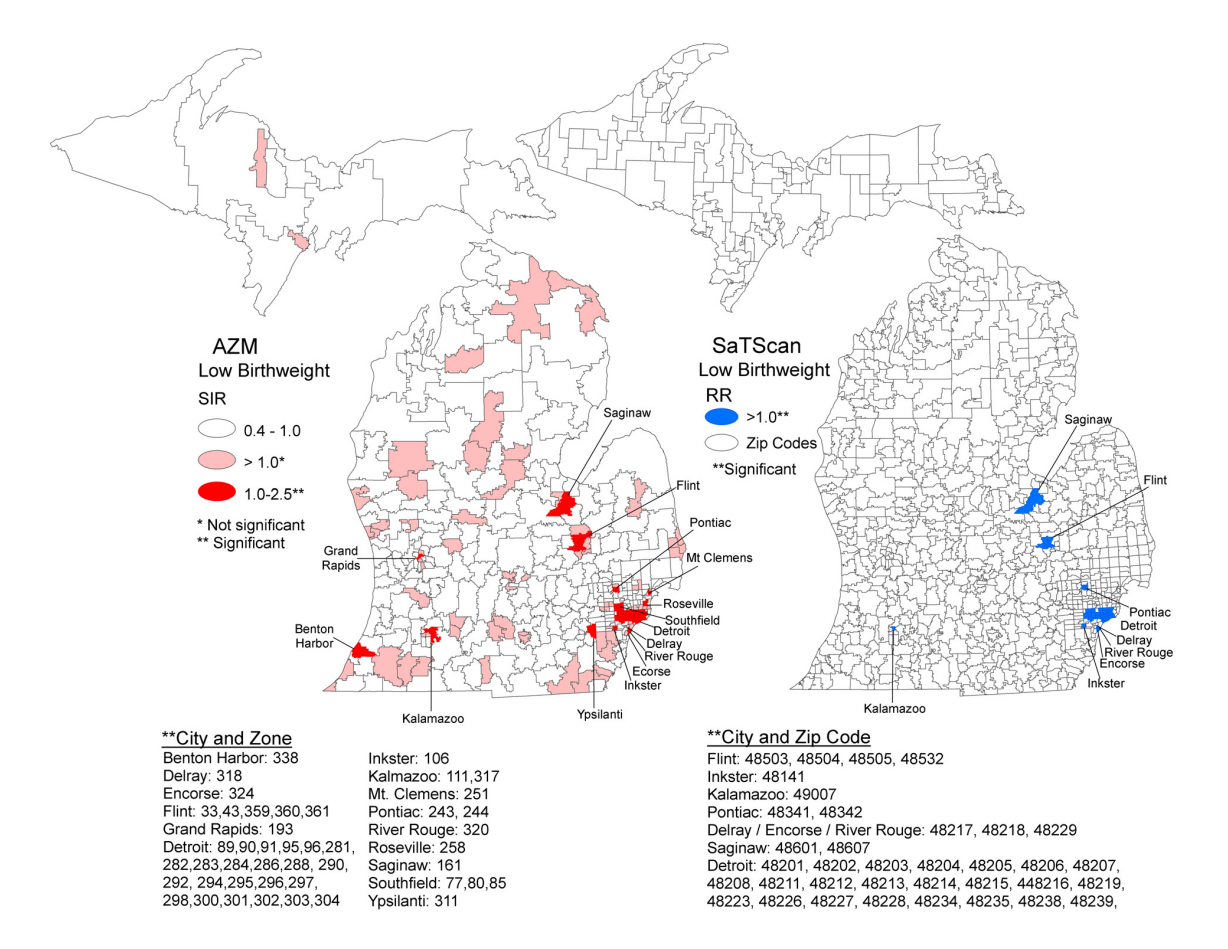
**Maps of low birthweight standardized incidence ratios following AZM compared with SaTScan clusters, Michigan 2004 to 2006**.

Figure 4 shows maps of SIRs for low birthweight estimated in AZM and significant clusters detected in SaTScan. Using AZM the spatial patterns of significantly high SIRs showed elevations in the major metropolitan principle cities, in order of significance, Detroit, Flint, Kalamazoo, Saginaw, Inkster, River Rouge, Encorse, Pontiac, Southfield, Mount Clemens, Grand Rapids, Benton Harbor and Ypsilanti. When these locations are compared with 7 significant SaTScan clusters there is congruency in order of significance Detroit, Flint, Saginaw, Inkster, River Rouge, Pontiac, and Kalamazoo. The two most significant clusters were in Detroit both comprising 23 Zip Codes. There were also a number of areas where AZM found significantly high SIRs in areas where there were no SaTScan clusters such as Benton Harbor, Encorse, Kalamazoo, Grand Rapids, Mount Clemens, Roseville, Southfield and Ypsilanti. Below the maps in Figure 4 are lists of cities and zones with elevated SIRs produced with AZM and cities and Zip Codes with clusters produced in SaTScan. These lists may be compared with Tables 2 and 4. Table 2 shows the low birthweight rates and SIRs with their 95% confidence intervals by zone. Table 4 shows the relative risk, log-likelihood ratio and p-value for each of the 7 SaTScan clusters.

**Table 4:** SaTScan clusters of low birthweight, Michigan 2002–2004.

Cluster	Relative Risk	Log-Likelihood Ratio	p-value
Detroit	2.0	543.0	0.001
Detroit	1.8	211.1	0.001
Flint	1.8	211.1	0.001
Saginaw	1.7	33.7	0.001
Inkster	1.7	18.9	0.001
River Rouge	1.7	17.8	0.001
Pontiac	1.5	15.2	0.001
Kalamazoo	2.0	14.4	0.001

**Figure 5:**
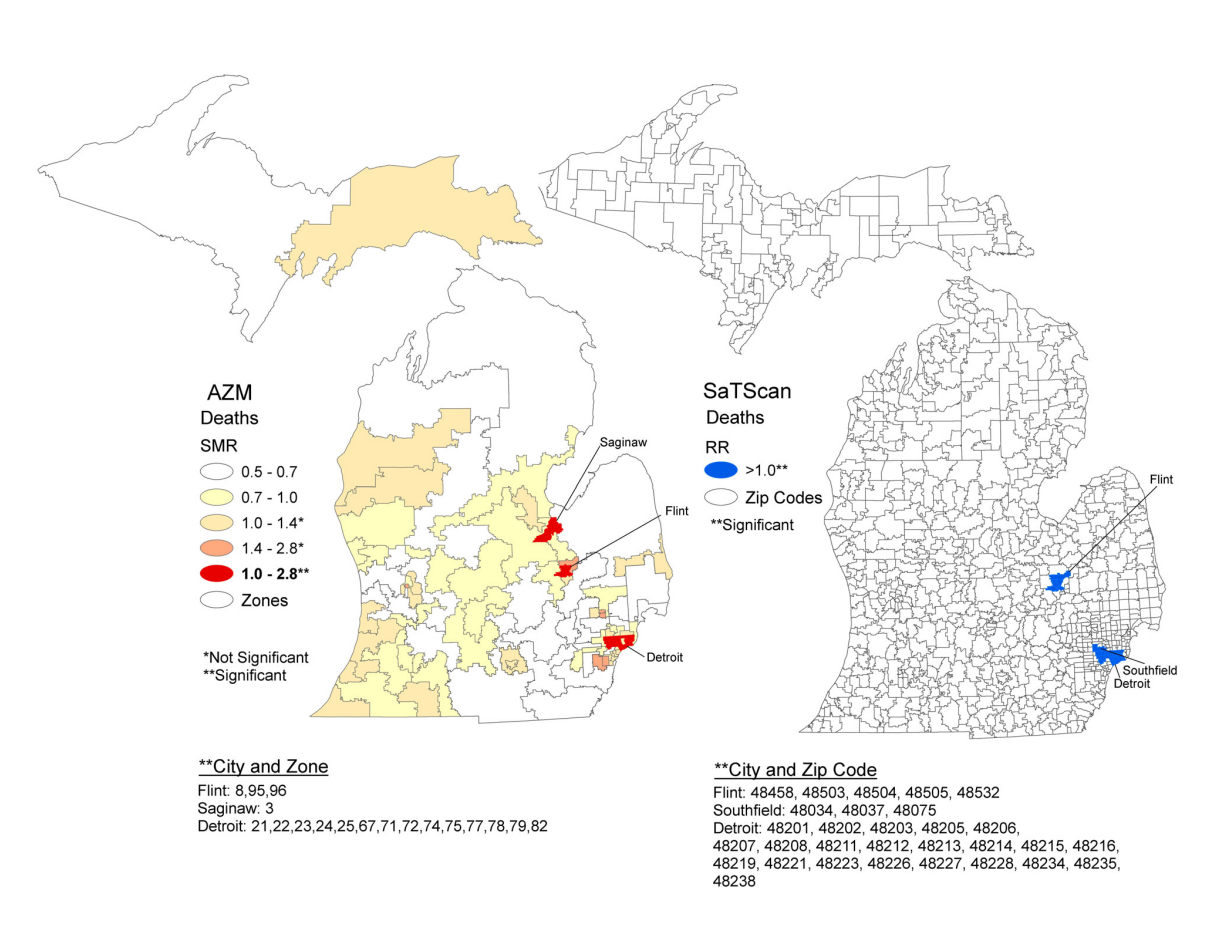
**Maps of standardized mortality ratios following AZM compared with SaTScan clusters, Michigan 2004 to 2006**.

Figure 5 shows maps of SMRs estimated in AZM and significant clusters estimated in SaTScan. Elevated SMRs were found in the metropolitan principal cities of Detroit, Flint and Saginaw. There were two significant SaTScan clusters in Detroit. The differences in infant mortality in Detroit between the two methods were two AZM zones not found in the Detroit clusters. One Detroit cluster also contained the suburb of Southfield that was not elevated using AZM. The elevated SMR in the Flint zone comprised four of the five Zip Codes found in the SaTScan significant cluster. SaTScan did not detect a cluster in Saginaw. Below the maps in Figure 5 are lists of cities and zones with elevated SMRs produced with AZM and cities and Zip Codes with clusters produced in SaTScan. These lists may be compared with Tables 3 and 5. Table 3 shows the infant mortality rates and SMRs with their 95% confidence intervals by zone. Table 5 shows the relative risk, log-likelihood ratio and p-value for each of the three SaTScan clusters.

**Table 5:** SaTScan clusters of infant deaths, Michigan 2002–2004.

Cluster	Relative Risk	Log-Likelihood Ratio	p-value
Detroit	2.2	71.7	0.001
Detroit*	1.8	35.7	0.001
Flint	1.8	33.7	0.001

*Cluster includes Southfield

## Discussion

Visualizing and exploring the spatial patterns of low birthweight and infant mortality rates in Michigan was an important first step in conducting a geographic evaluation of the State's reported high infant mortality rates. Using AZM we were able to calculate stable rates and ratios that would not have been possible using commonly used Zip Code boundaries. The spatial patterns derived from AZM showed areas throughout the state with elevated low birthweight rates. Elevated SIRs were primarily located in the principal metropolitan cities of Benton Harbor, Detroit, Flint, Kalamazoo, Saginaw, Inkster, River Rouge, Encorse, Pontiac, Southfield, Mount Clemens, Grand Rapids, Benton Harbor and Ypsilanti. In these cities African Americans comprise a large proportion of the population therefore these patterns also reflect spatial disparities in low birthweight by race in Michigan. These findings are consistent with previous research reporting high rates of adverse birth outcomes in racially segregated areas [28,31]. Importantly, there are other principal metropolitan cities that also have large African American populations that did not show significantly high rates of low birthweight or SIRs such as Battle Creek, Jackson and Lansing suggestive that race alone cannot completely explain the racial disparities in low birthweight observed in Michigan. Likewise, there are spatial disparities in infant mortality with Detroit, Flint and Saginaw having the highest death rates and other principal metropolitan cities that are also highly segregated by race but do not have significantly high infant death rates. Thus, in addition to studying spatial disparities by race in low birthweight and infant mortality, future research in Michigan should also investigate spatial differences within the African American population and potential mechanisms underlying their environments, including access to health care services.

The size of zones within the optimal zone designs is largely dependent upon the number of low birthweight births or infant deaths in Zip Codes. Low birthweight is not as rare an event as infant mortality; therefore, the size of zones created to meet the TP or minimum case threshold for low birthweight are smaller or more compact than zones created for infant deaths. In future studies these smaller zones may be useful for generating mechanistic hypotheses. For example, in this study we used maternal race as our homogeneity constraints, thus the zones in which low birthweight rates are calculated may be relatively homogeneous on this maternal characteristic in addition to being compact. Future studies of low birthweight that use environmental constraints may identify common risk factors associated with certain living environments. Previous studies [7,12] have shown the usefulness of AZM methodology to identify neighborhood social and built environmental risk factors for poor health outcomes as well as protective characteristics that lead to healthy living [16].

For infant mortality however, the size of the zones created to meet the TP or minimum case threshold for infant deaths were relatively large making it difficult to explore underlying mechanisms. Instead these zones may be better used to understand the demand for maternal and infant health care services, including neonatal-perinatal health care. Adding health care services as a homogeneity constraint will create zones that are homogeneous within (e.g., zones with high versus low utilization of services) and heterogeneous between on this constraint. Overlaying the health care services onto these zones may be useful in identifying areas that have services but lack utilization versus areas that do not have services but are in need. In future research we will also assess the need for health services within administrative units such as counties in case mother's who receive services in one county are unlikely to cross over into other counties. This research could utilize the capability of AZM to analyze multiple boundary definitions (e.g., Zip Codes and county boundaries) that were not investigated in this study. In Michigan the perinatal regionalization system was abolished in the mid-1980s due to budgetary constraints. There is an immediate need to evaluate the availability and accessibility of maternal and infant health care services for high-risk mothers and infants, including transport to neonatal intensive care units. This empirical example of AZM as a tool for public health surveillance could also be applied in the area of health services research.

**Figure 6:**
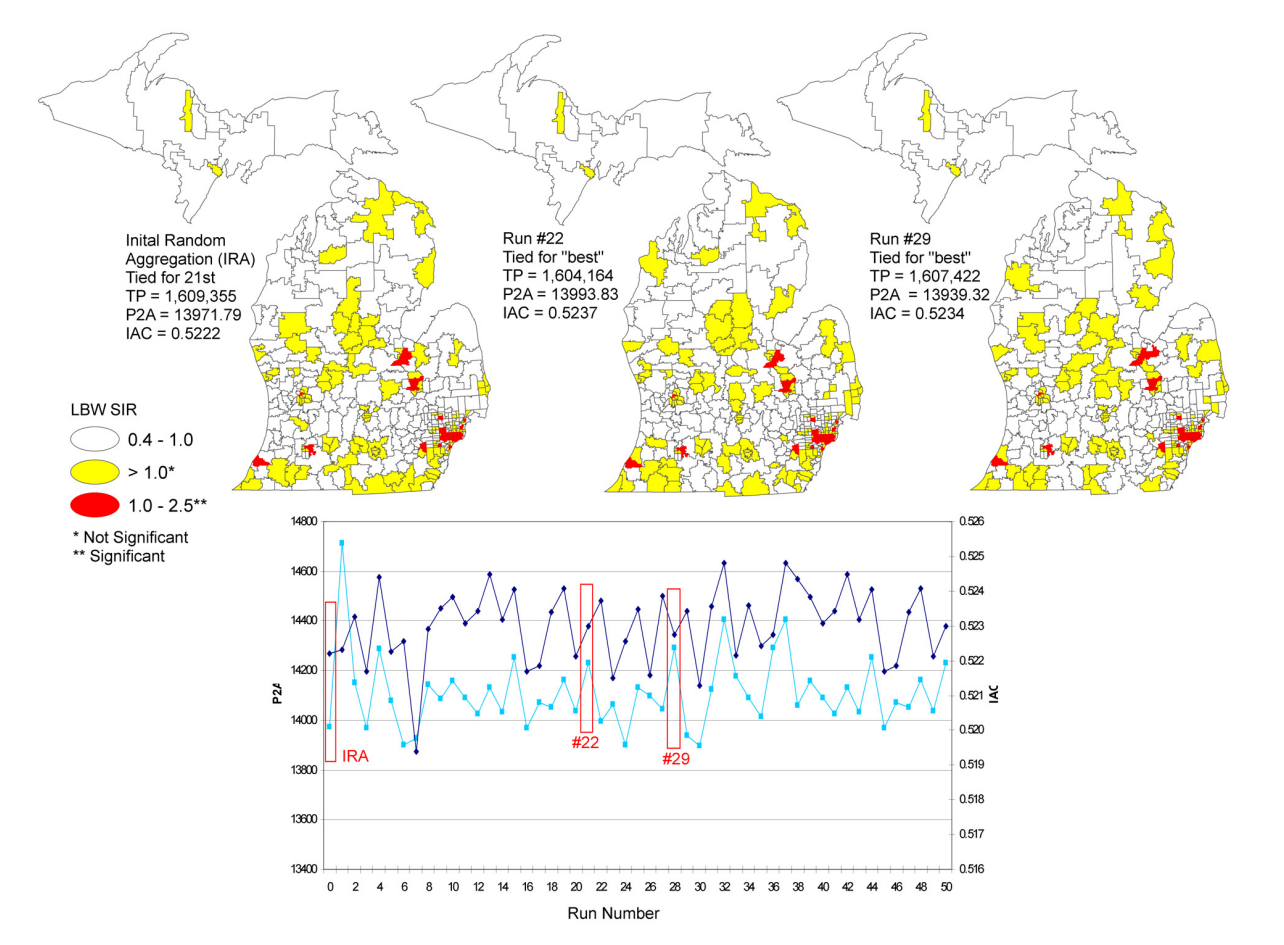
**AZM parameter constraint results for P2A and IAC following 50 restarts**.

The AZM parameters evaluated for optimal zone design in this study were the P2A and IAC. The TP output result was less important because all zones met the minimum case threshold criteria from within which to calculate stable rates and ratios. The evaluation included ranking the P2A and IAC output beginning with the IRA followed by 50 restarts each having 100 iterations. The IRA and the two runs with the least P2A and strongest IAC were selected as optimal zone designs. Thematic maps of low birthweight and infant mortality rates and SIRs and SMRs were constructed using these three zone designs (Figure 6).

In Figure 6 the IRA setting is shown as the first pair of points (i.e., P2A and IAC) on the far left-hand side of the graph. When AZM is run on multiple occasions using the same data, the same initial settings and the number of iterations, the same IRA will result in the same configuration of output zones. The subsequent 50 runs following the IRA resulted in slightly different sets of zone designs. We found two of these zone designs to be slightly more optimal than the IRA as shown in Figure 6 (runs 22 and 29); however, a comparison of the spatial patterns of SIRs for the three zone designs show all of the same significant areas in all thee maps. This sensitivity analysis demonstrates the stability of the spatial patterns (i.e., minimal MAUP zone effect) derived from AZM over time. There were virtually no differences except for one zone in the eastern portion of Saginaw. Therefore, we decided that for surveillance purposes the IRA run would be the ideal zone design to use because of its stable geography and the ability to monitor and evaluate spatial patterns of rates or ratios over time.

Finally, we conducted a comparative analysis of the output generated in AZM and SaTScan. The performance of AZM was very similar to that of SaTScan showing similar areas of elevated risk in SIRs and significant clusters. The AZM analysis identified additional areas with elevated SIRs that were not detected by SaTScan. We believe these differences were primarily due to the differences in modeling techniques. For example, AZM accounted for the homogeneity constraint "race" in the construction of zones thereby, forcing the clustering of women on this characteristic. Since the forced clustering was of women and not infants per se further investigation on the importance of this clustering on the low birthweight and infant mortality rates and ratios is warranted. SaTScan on the other hand has the ability to control for these individual-level risk factors (i.e., since we know that African American women are at increased risk of having a low birthweight infant SaTScan can remove the effect of race to detect clusters of women/infants in areas that are unknown). In this analysis we did not choose to remove the effect of race in SaTScan because it was used as an important constraint in AZM.

### Limitations

One limitation of our study is that while we tested the MAUP zoning effect on our rates and ratios we did not test the MAUP scale effect using different levels of administrative units. Future research should also apply AZM at the block group and census tract levels to test for the scale effect. Understanding the magnitude of the MAUP zoning and scale effects is important in validating the stability of the spatial patterns of rates and ratios. Since the focus of this study was to visualize and explore the spatial patterns of low birthweight and infant mortality we did not test mechanistic hypotheses with multilevel models. Multilevel models can be used to estimate the effect of individual characteristics within zones and area-level characteristics between zones. This analysis would be important since Stafford et al [16] found little differences between administrative and zoning boundaries in their health study. Relatedly, individual-level birth and infant death records were grouped to calculate rates and ratios and ecological fallacy occurs when analyses based on grouped data lead to conclusions different from those based on individuals. Therefore, the spatial patterns derived from this analysis may include aggregation bias due to the differential distribution of confounding variables created by grouping [34]. Using multilevel models in future research will help to decompose these rates and ratios by studying individuals nested within their environments. The sensitivity of zone designs on multilevel modeling results is an area for future research. Another limitation in our study was that some zones, especially for deaths were very large and if the purpose of future studies is to explore underlying risk factors for infant deaths in small areas then a Bayesian method would be preferred. Another limitation is that we used low birthweight as an outcome measure but future research should also study small for gestational age (SGA). SGA is defined as a birth weight less than the 10th percentile for a given gestational age based on a reference population of all singleton live births in the United States [35] and may be conceptually helpful in studying adverse birth outcomes in diverse populations. Finally, the Zip Code of mother's residence was used to map these spatial patterns of low birthweight and infant deaths but since a travel history was not available future research should also consider the movement of women and infants and their differential exposures during pregnancy up to death.

## Conclusion

This study showed that spatial patterns of low birthweight and infant mortality derived from using administrative boundaries such as Zip Codes can result in numerous errors across the state when the number of cases/deaths per unit is small. To correct this problem, researchers have used geographic methods such as probability mapping, spatial filters, also referred to as spatial smoothers, Bayesian smoothing techniques and cluster detection methods. This study demonstrates another technique called AZM tool for automated zone design to stabilize rates and ratios. AZM inputs boundaries but reconstructs the geography in areas with low case counts in order to increase case numbers to stabilize rates and ratios for thematic mapping. Thematic maps are a useful tool to visualize and explore spatial patterns because they are relatively easy to read and interpret from a research and laymen's (i.e., public) perspective. AZM is therefore, a valuable tool that could be used for geo-surveillance in public health departments, including the reporting of disease and health conditions to the public. From these spatial patterns specific zones of high risk can be identified from within which to study underlying mechanisms, target for monitoring and implementing public health intervention and prevention programs and evaluating the need for health care services.

Researcher's who wish to proceed with AZM however, need to be aware that the protocol to evaluate optimal zone design is largely subjective and dependent upon the study question, conceptual design of the study and input modeling parameters. Future research should continue to document conceptual frameworks of health studies in addition to the input modeling parameters to evaluate optimal zone design for different health outcomes.

## Methods

### Data

This study used vital statistics live birth and linked infant death records for the State of Michigan obtained from the Michigan Department of Community Health, Office of Vital Statistics and aggregated for the years 2004 to 2006 (n = 384,587). Only live singleton births were studied thus reducing the dataset to 370,588 births. The birth records were queried for low birthweight defined as infants born weighting less than 2,500 grams. Those birth records with birth weight less than 200 grams or greater than 6,000 grams were considered outliers and removed from the dataset (n = 211). There were 2,972 infant deaths during this time period, excluding those infant deaths that occurred in 2004 to infants born in 2003 and those infants who died in 2007 but were born in 2006. Infant deaths were defined as those infants born alive that died within their first year of life. The infant death records were linked to the live birth files using the birth certificate as the common identifier.

The Zip Code of mother's residence at the time of her infant's birth was the geographic unit of analysis. There were 201 birth records without Zip Codes that were removed from the dataset, further reducing the data to n = 370,386 records. The low birthweight, infant death and all birth records were aggregated by Zip Code and joined to a Zip Code boundary file produced by Environmental Systems Research Institute 2002 [36] (n = 896). Those Zip Codes whose boundary changed after 2002 were recoded to compliment the ESRI geographic Zip Code file. The recoding of Zip Codes was validated with the geocoded address of the mother and did not affect the original location-geographic position of the birth.

Since one of the purposes of our research was to evaluate AZM as a potential tool for public health surveillance we decided to utilize Zip Codes as the geographic unit of analysis because approximately 99% of birth records contained a Zip Code making these data readily available/timely for spatial analysis. Using the Zip Code as the unit of analysis also reduced potential bias associated with missing data. In this study, approximately 3% of records could not be geocoded (i.e., address matched) because the mother's address was given as a post office box number or a rural-route address. This potential bias would have been located largely in Upper Michigan. We recognize that Zip Codes may not reflect the local neighborhood environment in which mothers live and infants are born and that a finer resolution of geography is preferred to explore local hazards and potential exposures. To visualize and explore spatial patterns and to generate mechanistic hypotheses at a finer geographic scale the birth and linked infant death records would need to be geocoded and aggregated to the census block, block group or census tract. The authors decided that from a surveillance perspective, the Zip Code level of analysis would be conduced first while the records are being geocoded and thereafter, surveillance at a finer geographic scale could follow.

### Automated Zoning Methodology

Automated zoning was implemented using the Automated Zone Matching (AZM) 1.0.0 software written by David Martin [37]. This software is freeware and available for public use on David Martin's website [37]. This software incorporates the principles of automated zone design originally conceptualized by Oppenshaw [38]. In preparation for AZM analysis the ESRI Zip Code boundary file [36] was converted to an ArcInfo coverage and island polygons were removed and slivers and overshoots were removed and undershoots were corrected. After the topology was check and corrected the arc and polygon attribute information was exported for use in the AZM software. The arc files were uploaded in AZM as intersection and contiguity files. These files comprised the Zip Code geography used in subsequent analyses.

The first parameter selected was the population target (PT) and/or minimum population threshold constraint(s). As noted previously, we used 25 low birthweight cases or infant deaths as our ideal target and 20 cases/deaths as our minimum threshold. Thus, all new zones created had at least 20 cases/deaths from which to calculate stable rates (i.e., rates with RSE 20%, respectively). AZM functions by minimizing the squared difference between the target number of cases/deaths and the number of cases/deaths in each Zip Code [37]. Thus if every Zip Code contained exactly 25 cases/deaths this constraint would reach zero. The minimum population threshold was 50 births. This threshold was not specified in AZM but resulted after the aggregation of low birthweight cases/deaths. These TP parameters were held constant throughout the analysis.

The second parameter selected was the shape statistic defined as: $\sum q_{k}^{2}/A_{k}$

Where q_k _is the perimeter of zone k and A_k _is its area. AZM functions to minimize the perimeter squared divided by area, which maximizes shape compactness on zones. As outlined in Martin [37] software documentation "irregular shapes may have longer perimeters in relation to their area; thus, squaring the perimeter makes highly irregular shapes less attractive when this constraint is in operation."

The third parameter selected was the homogeneity constraint. The homogeneity constraint promotes homogeneity within zones and heterogeneity between zones by encouraging the aggregation of similar values. In this study we used mother's race (i.e., African Americans versus all others) as our homogeneity constraint. Maternal race was added because of the high levels of racial residential segregation in Michigan's cities and the desire to capture spatial-racial disparities in low birthweight and infant mortality. The IAC for these two racial groups was obtained for each k category and overall K across all categories (k1, k2 = K categories). An IAC of 0.5 implied a reasonable degree of homogeneity [37].

The IAC was calculated as:

$$\delta_{k}=\frac{\frac{1}{M-1}\sum_{g=1}^{M}N_{g}{\left(P_{kg}-P_{k}\right)}^{2}}{\left(\bar{N}*-1)P_{k}(1-P_{k}\right)}-\frac{1}{\left(\bar{N}*-1\right)}$$

Where, $\bar{N}*$ was the mean case/death size of the M number of Zip Codes, with an adjustment [39] to take into account variation in the case/death size of units. It was expected that $\bar{N}*$ would be very close to $\bar{N}$. *N*_*g *_was the case/death size of Zip Code g; *P*_*k *_was the overall proportion of cases/deaths in category k, and *P*_*kg *_was the proportion in category k in Zip Code g. Thus, the IAC was approximately the ratio of the Zip Code variance to the maternal level variance, and this ratio was divided by the mean case/death size.

After *δ*_*k *_was calculated for each race k category the overall IAC, *δ *for all categories was calculated as:

$$\text{IAC }=\frac{1}{k-1}\sum_{k=1}^{K}(1-P_{k})\delta_{k}$$

An optional parameter that may be changed for user preferences was the "random number initialization value," which sets the seed value for the pseudorandom number generator prior to the IRA run. Keeping this value constant during multiple restarts of AZM would result in the same zone designs. Changing this seed value would result in a different sequence of pseudorandom decisions, which would alter the zone designs. For the purposes of this surveillance research we kept this parameter constant to eliminate differences between zone designs. With these model parameters the AZM analysis was conducted by running 50 program restarts with 100 iterations each taking the run (i.e., zone design) with the most compact shape, the strongest IAC and lastly the best TP statistic.

Following this reconstruction of zones the rates and ratios were calculated for each zone using SAS 9.03 software [40]. The incidence rates were calculated as the number of low birthweight births divided by the total number of births * 100. The relative standard errors (RSE) and 95% confidence intervals (95% CI) for the incidence rates were calculated using the methodologies [3,4] described below.

The RSE for the low birthweight incidence rates were calculated as:

$$\frac{rate}{\sqrt{cases}}\times \frac{1}{rate}\times 100=\frac{1}{\sqrt{cases}}\times 100$$

A binomial distribution was used to estimate the 95% CI for the low birthweight incidence rates when the number of low birthweight cases was greater than or equal to 100:

$$\text{Lower limit }=rate-[1.96\times rate/\sqrt{births}]$$

$$\text{Upper limit }=rate+[1.96\times rate/\sqrt{births}]$$

A Poisson distribution was used to estimate the 95% CI for the low birthweight incidence rates when the number of low birthweight cases was less than 100:

Lower limit = *rate *× *L*

Upper limit = *rate *× *U*

Where *L*(0.95, *rate*)and *U*(0.95, *rate*)are values that correspond to the number of births provided in NVSR [3].

The RSE and 95% CI for the infant mortality rates were calculated using the methodologies [3,4] described below.

The RSE for the infant mortality rates were calculated as:

$$\sqrt{\frac{1}{deaths}+\frac{1}{births}}\times 100$$

A binomial distribution was used to estimate the 95% CI for the infant mortality rates when the number of births was greater than or equal to 100:

$$\text{Lower limit }=IMR_{i}-(1.96\times IMR_{i}\times \frac{RSE(IMR_{i})}{100})$$

$$\text{Upper limit }=IMR_{i}+(1.96\times IMR_{i}\times \frac{RSE(IMR_{i})}{100})$$

A Poisson distribution was used to estimate the 95% CI for the infant mortality rates when the number of births was less than 100:

Lower limit = *IMR*_*i *_× *L*(0.95, *D*_*adj*_)

Upper limit = *IMR*_*i *_× *U*(0.95, *D*_*adj*_)

where, *D*_*adj *_was the adjusted number of infant deaths (rounded up to the nearest integer) used to take into account the RSE of the number of infant deaths and live births, and was computed as follows:

$$D_{adj}=\frac{Deaths\times Births}{Deaths+Births}$$

*L*(0.95, *D*_*adj*_) and *U*(0.95, *D*_*adj*_)refer to limits provided in NVSR [3].

Indirect standardization was used to calculate unadjusted low birthweight SIRs and infant deaths SMRs. The standardized morbidity ratios were calculated by dividing the observed number of low birthweight cases *O*_*i *_by the expected number of cases (*E*_*i*_) for each zone. The expected numbers of cases were derived using the formula:

$$E_{i}=\frac{b_{i}}{B_{i}}\times Zone_{i}$$

where: *b*_*i *_= number of low birthweight births in Michigan for the years 2004–2006

*B*_*i *_= number of live births in Michigan for the years 2004–2006

*Zone*_*i *_= number of live births in a zone

Ninety-five percent confidence intervals were also calculated for the standardized incidence and mortality rates.

Following the calculation of the rates and ratios by zone these data were rejoined to the zone geography, input into ArcGIS 9.2 [36] and thematic maps were created. The spatial patterns derived by AZM were validated by comparing the location of zones with high SIRs and SMRs with statistically significant spatial clusters of low birthweight or infant mortality derived in SaTScan. SaTScan is cluster detection software developed by Dr. Martin Kulldorff. It is freeware and publically available at the website: [41]. SaTScan has been used in many health studies as a surveillance tool to explore clusters of disease in space, time and space-time [41]. In this study we detected only spatial clusters by scanning a circular/ellipse window across the centroids of all Zip Codes noting the number of observed and expected low birthweight cases or infant deaths inside the window at each location. We used a Poisson-based model, where the number of low birthweight cases or infant deaths in each Zip Code was assumed to be Poisson distributed, according to the underlying births at risk. The expected number of cases in each Zip Code was calculated as:

*E*[*c*] = p* *C*/*P*

Where c was the observed number of cases and p was the number of births in the Zip Code of interest, while C and P were the total number of cases and births respectively. A relative risk was derived by dividing the observed number of cases by the expected number of cases.

The alternative hypothesis is that there was an elevated risk within the window as compared to the outside. Under the Poisson assumption, the likelihood function for a specific window was proportional to:

$${\left(\frac{c}{E\left[c\right]}\right)}^{c}{\left(\frac{C-c}{C-E\left[c]\right]}\right)}^{C-c}I()$$

Where C was the total number of cases, c was the observed number of cases within the window and E [c] was the adjusted expected number of cases within the window under the null-hypothesis. C-E [c] was the expected number of cases outside the window. I () was an indicator function. When SaTScan scans for clusters with high rates, I () was equal to 1 when the window had more cases than expected under the null-hypothesis, and 0 otherwise [41]. Hypothesis testing was conducted using 999 Monte Carlo simulations. Finally a test statistic was calculated for each random replication as well as for the real dataset and if the latter was among the 5% highest then the test was significant at the 0.05 level. In this study we scanned for clusters of geographic sizes that would capture between zero and 6.4% of births at risk for low birthweight, which represented the statewide rate of low birthweight. We also scanned for clusters of geographic sizes that would capture between zero and 7.6% of births representing the statewide infant mortality rate. There was no geographic overlap in clusters.

## Competing interests

The authors declare that they have no competing interests.

## Authors' contributions

SCG originated the study and participated in the planning of the study, analysis of the data and writing of the brief. HE contributed to the preparation of the data, analysis of the data, and writing of the brief.

## References

[B1] Michigan Department of Community Health. http://www.michigan.gov/mdch/0,1607,7-132-2944_4669---,00.html.

[B2] Martin D, Nolan A, Trammer M (2001). The application of zone-design methodology in the 2001 UK Census. Environment and Planning A.

[B3] National Vital Statistics Reports (2007). Births: Final Data for 2005. Centers for Disease Control and Prevention.

[B4] Healthy People 2010 Statistical Note (2002). Healthy people 2010 criteria for data suppression.

[B5] US Census Bureau (2002). Source and accuracy of the data for the March 2001 current population survey microdata file, 2001. National Vital Statistics Reports.

[B6] Library of Michigan, Department of History, Arts, and Libraries (2003). Metropolitan and Micropolitan Statistical Areas in Michigan based on the 2000 Census.

[B7] Cockings S, Martin D (2005). Zone design for environment and health studies using pre-aggregated data. Social Science and Medicine.

[B8] Openshaw S (1984). The Modifiable Areal Unit Problem CATMOG 38.

[B9] Haynes R, Daras K, Reading R, Jones A (2007). Modifiable neighbourhood units, zone design and residents' perceptions. Health and Place.

[B10] Daras K, Haynes R, Daras K, Reading R, Jones A (2007). An information statistics approach to zone design in the geography of health outcomes and provision. PhD Thesis Modifiable neighbourhood units, zone design and residents' perceptions Health and Place.

[B11] Openshaw S, Rao L (1995). Algorithms for reengineering 1991 census geography. Enviornment and Planning A.

[B12] Flowerdew R, Manley DJ, Sabel CE (2008). Neighbourhood effects on health: Does it matter where you draw the boundaries?. Social Science and Medicine.

[B13] Martin D (2003). Developing the automated zoning procedure to reconcile incompatible zoning systems. International Journal of Geographical Information Science.

[B14] Stafford M, Duke-Williams O, Shelton N (2008). Small area inequalities in health: Are we underestimating them?. Social Science and Medicine.

[B15] Alvanides S, Openshaw S, Rees P, Rees P, Martin D, Williamson P (2002). Designing your own geographies. The Census Data System.

[B16] Riva M, Apparicio P, Gauvin L, Brodeur J (2008). Establishing the soundness of administrative spatial units for operationalising the active living potential of residential environments: An exemplar for designing optimal zones. International Journal of Health Geographics.

[B17] Waller LA, Gotway CA (2004). Applied spatial statistics for public health data.

[B18] Rushton G (2003). Public health, GIS and spatial analytic tools Annual Review of Public Health.

[B19] Talbot TO, Kulldorff M, Forand SP, Haley VB (2000). Evaluation of spatial filters to create smoothed maps of health data. Statistics in Medicine.

[B20] Johnson GD (2004). Small area mapping of prostrate cancer incidence in New York State (USA) using fully Bayesian hierarchical modeling. International Journal of Health Geographics.

[B21] Forand SP, Talbot TO, Druschel C, Cross PK (2002). Data quality and the spatial analysis of disease rates: Congenital malformations in New York State. Health and Place.

[B22] Ozdenerol E, Williams BL, Kang SY, Magsumbol MS (2005). Comparison of spatial scan statistic and spatial filtering in estimating low birth weight clusters. International Journal of Health Geographics.

[B23] Boyle MH, Willms JD (1999). Place effects for areas defined by administrative boundaries. American Journal of Epidemiology.

[B24] Glaster G (2001). On the nature of neighbourhood.. Urban Studies.

[B25] Picket K, Pearl M (2001). Multilevel analyses of neighbourhood socioeconomic context and health outcomes: a critical review. Journal of Epidemiology and Community Health.

[B26] Diez Roux AV (2001). Investigating neighborhood and area effects on health. American Journal of Public Health.

[B27] Macintyre S, Ellaway A, Cummins S (2002). Place effects on health: How can we conceptualise, operationalise and measure them?. Social Science and Medicine.

[B28] Kawachi I, Berkman LF (2003). Neighborhoods and health.

[B29] Oaks JM (2004). The (mis)estimation of neighborhood effects: causal inference for a practicable social epidemiology. Social Science and Medicine.

[B30] Diez Roux AV (2004). Estimating neighborhood effects: the challenges of causal inference in a complex world. Social Science and Medicine.

[B31] Grady SC (2006). Racial disparities in low birthweight and the contribution of residential segregation: A multilevel analysis. Social Science and Medicine.

[B32] Rauh VA, Landrigan PJ, Claudio L (2008). Housing and health; intersection of poverty and environmental exposures. Annals New York Academy of Science.

[B33] National Vital Statistics Reports (2007). Infant Mortality Statistics from the 2004 Period Linked Birth/Infant Death Data Set. Division of Vital Statistics.

[B34] Cromley EK, McLafferty SL (2002). GIS and public health.

[B35] Alexander GR, Himes JH, Kaufman RB, Mor J, Kogan M (1996). A United States national reference for fetal growth. Obstetrics & Gynecology.

[B36] Environmental Systems Research Institute (ESRI). http://www.esri.com/.

[B37] Martin D David Martin’s Software Automated Zone Matching. http://www2.geog.soton.ac.uk/users/martindj/davehome/software.htm.

[B38] Openshaw S (1977). A geographical solution to scale and aggregation problems in region-building, partitioning and spatial modeling. Transactions of the Institute of British Geographers, New Series.

[B39] Tranmer M, Steel D (1998). Using census data to investigate the causes of the ecological fallacy. Environment and Planning.

[B40] SAS Institute Inc., SAS 9.1.3. http://www.sas.com.

[B41] Kulldorff M SaTScan. http://www.satscan.org/.

